# Perspective on Electrochemical Lead Detection: Distributed Sensing to Address Unevenly Distributed Risk

**DOI:** 10.1149/2754-2726/aeaf67

**Published:** 2026-10-13

**Authors:** Elizabeth R. Jacobia, A. Andrew D. Jones III

**Affiliations:** 1 Department of Civil & Environmental Engineering, Duke University, Durham, NC 27708, United States of America

## Abstract

Lead contamination is a global issue and requires detection for risk evaluation and remediation. Standardized testing is accurate and effective for water quality monitoring, however, does not provide real-time results and is not widely accessible. Electrochemical detection methods have been developed with comparable lead sensitivity and selectivity to standard methods. United States Environmental Protection Agency certification of electrochemical lead detection methods and inspectors would allow for readily available and cost-efficient water quality results. Introducing electrochemical lead detection methods in the United States will increase water quality awareness, incentivize mitigation, and provide data for regulation and policy development.

## Highlights

Electrochemical lead sensors support at-home detection like EPA-approved paint tests.
Electrochemical lead sensors can be an integral part of the water smart grid.
Electrochemical lead sensors support lab methods for real-time detection.
Electrochemical lead sensors must overcome sample preparation and robustness limits

## Graphical abstract

**Figure:**
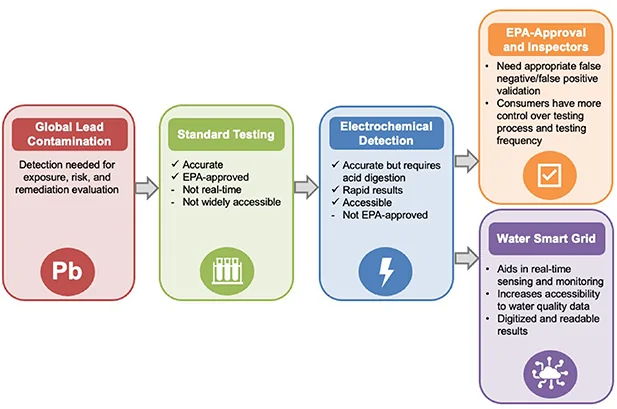

Lead is a neurotoxin that affects brain development in fetuses and children subclinically-by causing medical conditions that may not produce obvious symptoms.^1^ Lead toxicity was initially assumed to only cause clinical medical conditions at extremely high exposures, including heart disease, high blood pressure, kidney failure, reduced red blood cell production, joint and muscle pain, convulsions, and death.^2^ Currently, lead contamination is understood to impact people of all ages, including fetuses. For example, high lead exposure has been associated with low birth rates and fetal death.^3^ Waldron and Hanna-Attisha et al. describe human health impacts of lead exposure over time in more depth.^1^^,^^4^ It has been determined that there is no safe level of lead exposure.^5^^–^^7^ Despite early recognition of lead toxicity, reducing lead exposure has been challenging in the United States (US).^8^^,^^9^ Kim et al. (1999), Ores (2019), and Marcus (2026) describe the evolution of regulations to combat elevated lead levels and overall human exposure.^8^^–^^10^ Currently, the United States (US) maximum lead level goal is 0 mg ml^−1^ and the enforceable US Environmental Protection Agency (EPA) action limit is 10 *µ*g l^−1^. However, the US action limit is above the European Union and Canada lead action limit set at 5 *µ*g l^−1^ .^6^^,^^7^^,^^11^

Common sources of lead exposure include gas emissions near highways and airports, lead in soil and food, leaded paint chips, lead dust, and drinking water, all with the potential to cause subclinical negative health conditions.^2^ In the US, an estimated 50%–75% of lead contamination in drinking water is associated with lead service lines (LSLs), the line connecting a building to the utility water main (Fig. 1).^12^ Additional sources of lead in drinking water includes brass fittings, premise plumbing, galvanized pipes that accumulated lead scale, and lead solder.^12^^,^^13^ Approximately 13% of the US population get their water from unregulated private wells which may contain leaded plumbing and lack corrosion control treatment.^14^ The Lead and Copper Rule Improvements (LCRI) sets a US national goal and deadline for identifying and replacing all LSLs by 2037.

**Figure 1.**
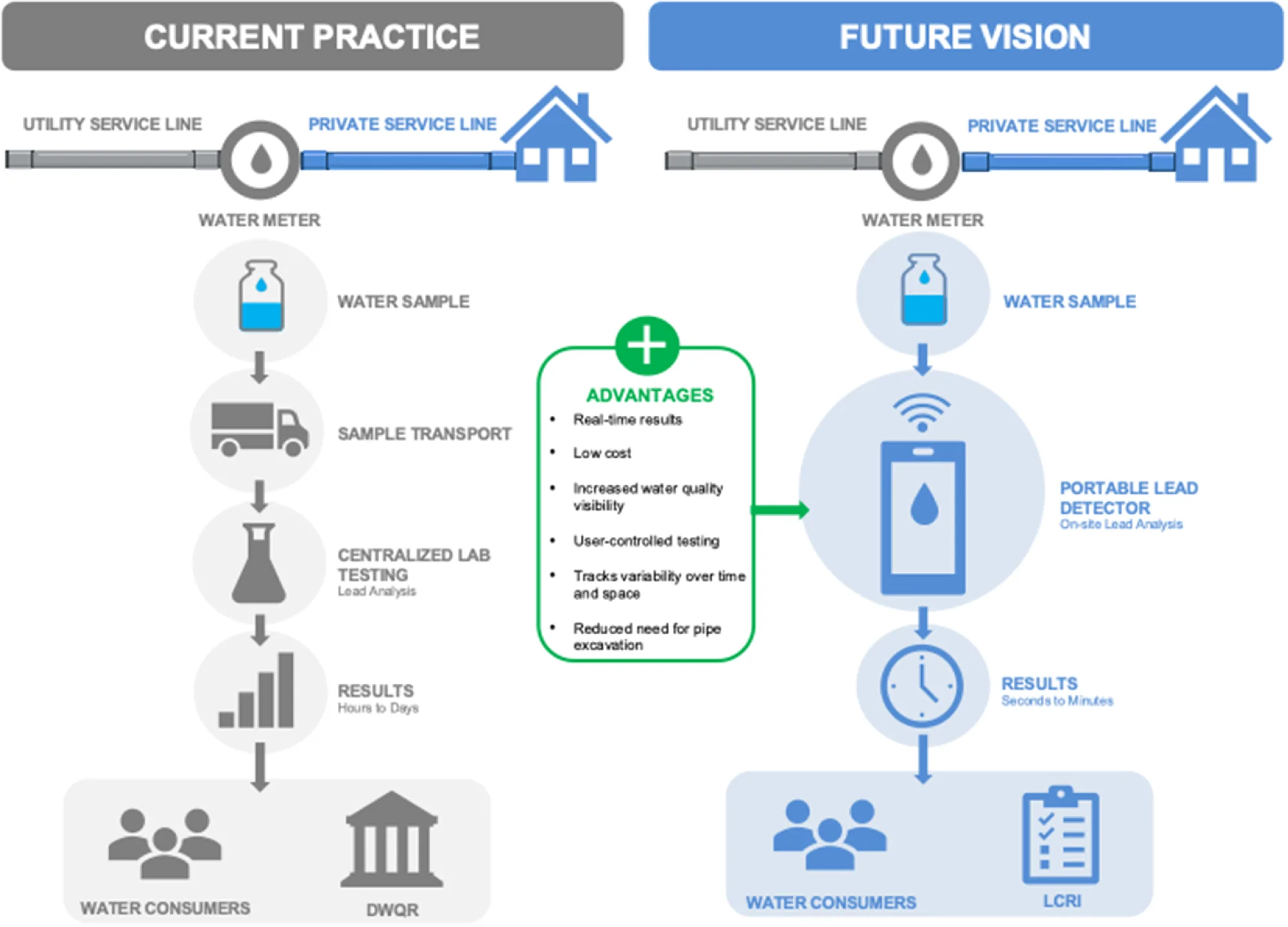
Utility and Private Service Line Detection Process Scheme. Current lead detection practices for utilities requires water sampling, sample transport to centralized labs for testing, and analysis processing for water consumers and the Drinking Water Quality Report (DWQR). The future vision for drinking water testing could be sample collection, a portable lead detector with instantaneous results used to inform water consumers and the LCRI. Portable lead detectors provide various advantages compared to lab-based detection techniques.

The Drinking Water State Revolving Fund and Water Infrastructure Finance Innovation Act support LSL removal and replacement, treatment system upgrades, detection system upgrades, and aid in the LCRI goal to inventory and remove all LSLs by 2037. The EPA is responsible for delegating Drinking Water State Revolving Fund allocations and Congress directly appropriates support using the Water Infrastructure Finance Innovation Act for longer term water system management. Though these resources seem useful, funds are difficult to secure and often not reserved for private homeowners. The Drinking Water State Revolving Fund and Water Infrastructure Finance Innovation Act projects can be informed and verified by water quality testing.

As lead plumbing corrodes and becomes soluble, it leaches into the drinking water system. Soluble lead has been controlled and is required to be controlled by phosphates, carbonates, or silicates, also known as optimal corrosion control (40 CFR 141.82). These chemicals create insoluble lead scale on plumbing to reduce lead release into the water. Accumulated scale has the potential to flake off as particulate lead or dissolve under the right conditions and contaminate the water.^15^ Point-of-use (POU) filters are recommended as a temporary solution to mitigate lead particulates under the LCRI and have been implemented to reduce lead consumption and therefore exposure at the tap.^16^ LSL replacements have been shown to cause lead scale disruption and therefore increased contamination of the drinking water supply.^17^ Mobilized lead also has the potential to settle on plumbing that did not originally have lead, creating persistent contamination even without an explicit lead source.^17^ Additional contamination due to various chemical and physical factors further emphasizes the need for increased water quality testing. Lead detection can be used to measure human exposure, effectiveness of treatment techniques, and lead scale mobilization because of the unpredictable complexity and variability of water systems.

## The Lead Days are not Over: The Current Social State of Lead in the US

The crises in Washington DC (2000–2004) and Flint, Michigan (2014–2016) are extreme examples of changing water conditions that caused major water quality disruptions and further mistrust within already socially, politically, and economically marginalized communities.^18^ Both events were due to treatment changes made by the town/utility to save costs that changed the drinking water chemistry, releasing high lead levels into the water supply. Consumers were exposed to elevated levels of lead over an extended period. The crises in Washington DC and Flint, MI were not failures of regulations or detection, but failures of incentives and oversight.^17^ While the regulations for optimal corrosion control, quality control measurements, and reporting were in place, systematic structures were lacking, allowing for reporting of false lead levels.^17^ For more detailed breakdown of the failures, see the review by Roy and Edwards.^17^

The Flint and Washington DC water crises inspired research, legislative, and regulatory revisions. The EPA is now empowered to sanction states’ environmental agencies and drinking water systems if they reasonably prove violations like what occurred in Flint, MI (40 CFR 141.82(i) and 40 CFR 141.83(b)(7)). However, in the 10 years since Flint, a news database search (Proquest, 2026-04-) for “lead in drinking water” showed reporting on lead exceeding action levels in Elgin, IL (a suburb of Chicago) in 2025; in 25% of licensed daycares in California in 2023; in schools in Maine and Delaware in 2022; in 1350 schools in Illinois in 2023, and schools in Boston, MA and San Diego, CA in 2018.^19^ Even noting that children are the most vulnerable to the effects of lead, places such as New Jersey public schools and daycare systems operate with low financial margins, therefore cannot test adequately and often shut-off faucets identified as lead-contaminated instead of replacing the leaded plumbing.^20^^,^^21^ At best, this creates two-tiered childcare and education systems, ones with funding for new and fully renovated construction and ones without. At worst, this leaves children as the sensor for detecting lead during routine pediatric visits.^4^

Lead detection methods are needed to inform both the LCRI and water consumers (Fig. 1). The coverup in Flint, MI in particular increased distrust in government institutions and the acceptance of misinformation.^22^^,^^23^ Non-routine and routine testing aids in water quality visibility, improving trust, and accounts for temporal and spatial variability.^22^^,^^23^ Due to the cost and time limitations of LSL replacements, detection can provide useful insight into lead pipe location and the extent of the contamination. Identifying problem areas and overall risk helps determine priority zones for action and remediation. Detection can inform and incentivize communities to push for better water testing, funding, and remediation resources.^24^ Until total elimination of the lead source, both centralized and decentralized testing are necessary to detect and prevent harm. Routine and spontaneous detection helps shift away from reactive lead testing and moves towards proactive intervention. More water quality data can inform policies, regulations, funding allocations, and risk evaluations to protect human health.^25^

## Current detection methods

Current lead detection methods such as EPA Method 200.8 and EPA Method 200.7 use inductively coupled plasma-mass spectrometry (ICP-MS) and inductively coupled plasma-optical emission spectroscopy (ICP-OES) because of their high sensitivity and trace analysis.^26^^,^^27^ Atomic absorbance spectroscopy (AAS) is another highly sensitive standard method for lead detection in drinking water. Appropriate preservation, digestion, and sample handling are essential when the analytical objective is total lead, including dissolved and particulate fractions (Fig. 1). Standard sample preparation methods in EPA Method 200.8 and 200.7 use nitric acid to lower pH below 2 for improved lead recovery, reduced matrix effects, and high capability with lead detection methods.^28^ Nitric acid (pH < 2) is able to digest multiple lead forms common in drinking water such as lead carbonates, lead phosphates, lead oxides, and solder.^29^ Hydrochloric acid (pH < 2) is used as a sample preparation additive to improve digestion, however, is not the primary acid treatment due to interferences with standard detection methods.^26^^,^^27^ Standard detection methods for lead in drinking water are laboratory-based, expensive, and require trained personnel. The cost of running these instruments ranges from $0.29—$0.66 per sample assuming 20 000 samples/year not including capital equipment, maintenance, service, certification costs or salaries.^30^ In addition to being cost-prohibitive for many municipalities, home inspection companies, and mobile home parks in charge of their own water, these costs are not scalable to provide comprehensive coverage in space or time. While these methods are sensitive and standardized, they also cannot provide real-time or high spatial resolution results, missing large exposure events like the Washington, DC crises.

Miniaturized mass spectrometry, colorimetry, fluorometry, and biosensing are being developed as portable and inexpensive metal detection methods. While these alternative methods show promise, they have not been implemented into standard practice. Miniaturized mass spectrometers have been in development for environmental testing applications. The goal was to reach accuracy comparable to their benchtop counterparts, however, success has been limited.^31^ Colorimetric methods such as at-home lead test strips have been promoted as simple, easy, and cheap lead indicators and are widely accessible. They are user friendly due to direct measurement of the water sample, however, they are not quantifiably accurate.^31^^,^^32^ Fluorescence handheld devices are feasible for field applications but are unaffordable to the average homeowner and not in widespread use amongst environmental testing services.^31^ Commercialized handheld devices include the HF-38 Fluorimeter (HF Scientific, FL, USA) and the VipIR (908 Devices, MA, USA). Biosensors have been developed to account for the cost limitations and sensitivity of the other detection methods. While they are a good cost alternative, biosensors vary in sensitivity and reliability.

## Electrochemical Sensors: Past & Present

 Electrochemical sensors can be portable, affordable, sensitive, selective, and easy to interpret, but are application dependent.^33^^,^^34^ Electrochemical lead detection publication numbers have rapidly increased over the past 45 years. Many of these sensors target media, such as blood and were designed to operate in laboratory settings. While the trend is similar, electrochemical lead detection for drinking water specifically shows only 201 publications with increases near national public crises like Flint, MI and Washington DC (Fig. 2). Though electrochemical sensors can be limited by more expensive analytical supporting equipment, cheaper supporting equipment is being developed (Fig. 2). Differential pulse stripping voltammetry (DPSV), anodic stripping voltammetry (ASV), and square wave stripping voltammetry (SWSV) are commonly used preconcentration and readout approaches for metal analysis. Electrochemical lead detection was initially achieved using hanging drop mercury electrodes.^35^ Later work used electrode materials such as screen printed gold electrodes, glassy carbon, copper, reduced graphene oxide, a NOx organic hybrid WOX–ethylene-diamine (WOX–EDA) nanowire, Fe/Mg/Ni-ternary lactate dehydrogenase, and boron doped carbon.^36^^–^^39^ Bismuth coating of glassy carbon and boron doped carbon is more sensitive than carbon substrates alone due to bismuth-lead alloy formation, encouraging the reduction of lead(II) to lead(0).^40^ Bismuth-based electrode are advantageous because they provide discriminating peaks for multiple heavy metals, are not sensitive to dissolved oxygen, more environmentally friendly than standard mercury electrodes, and non-toxic.^41^

**Figure 2.**
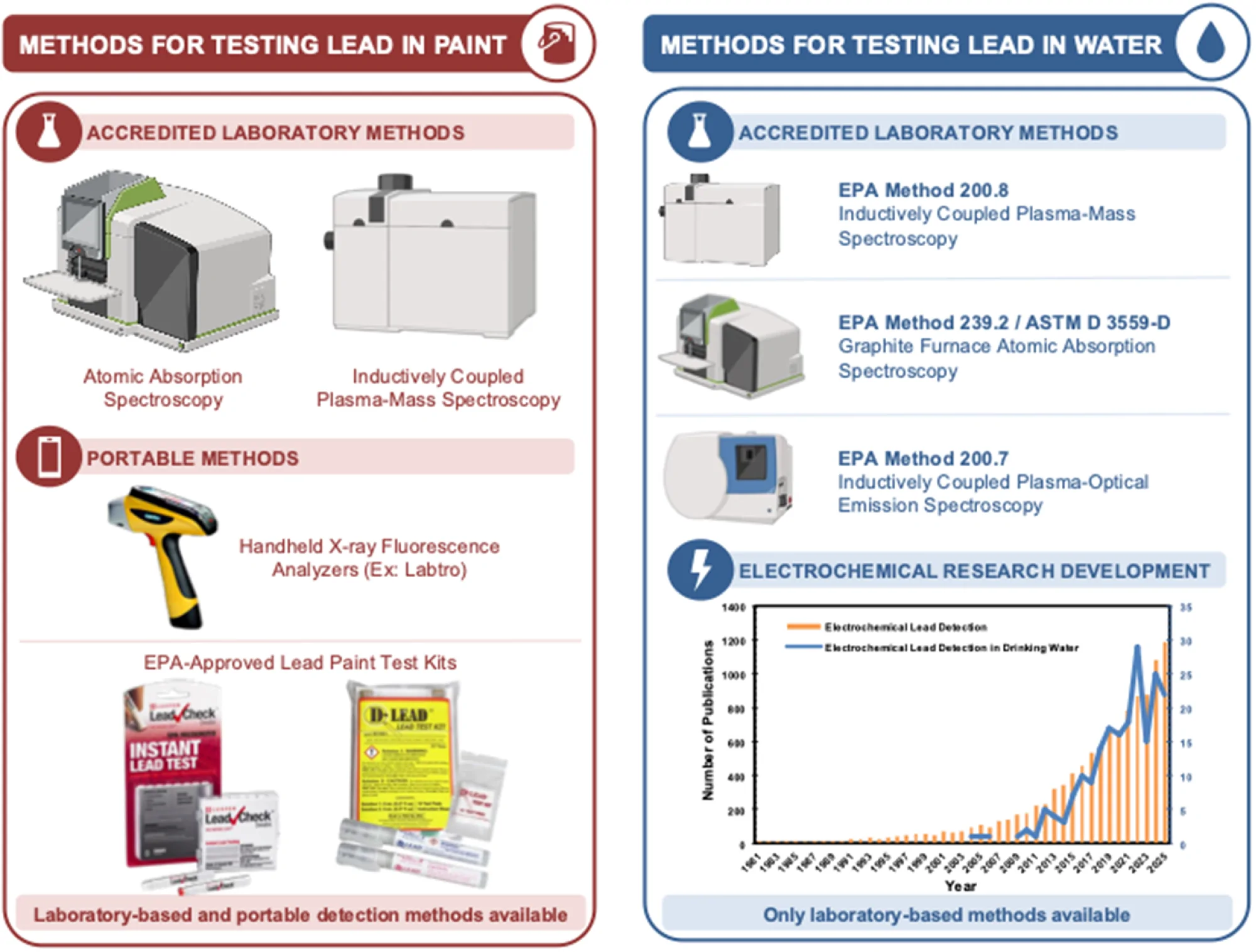
Methods for Testing Lead Paint vs Methods for Testing Lead in Drinking Water. Options for testing lead in paint include both accredited laboratory methods and portable methods such as handheld X-ray fluorescence analyzers and EPA-approved lead test kits. Accredited laboratory methods are the only government-recognized methods available for testing lead in drinking water. A Web of Science literature search showed an increase in general development (orange, n = 10,768, “electrochemical lead detection*”) and water-specific development (blue, n = 201, “electrochemical lead detection* and drinking water*”) of electrochemical lead detection methods over the past 45 years.

Screen printed electrodes (SPEs) are disposable, low-cost electrochemical sensors that have been developed for portable detection applications. SPEs can be modified to target specific analytes of interest. For example, bismuth modifications are often performed on carbon-based SPE substrates for heavy metal sensing. Fakayode et al. (2023) summarized (cf Fig. 1, Fakayode et al., 2023) the vast applications and high sensitivity capabilities of recently developed SPEs for trace heavy metal detection.^42^ While SPEs show promise, most developed sensors have been limited to lab-scale use. There has been a push to implement SPEs into real world applications to improve efficiency and cost of environmental testing. Other disposable bismuth-based electrodes for heavy metal sensing include paper-based electrodes and conductive carbon tape. Bismuth-modified SPEs (0.0008 *µ*g ml^−1^) and conductive carbon tape (0.002 *µ*g ml^−1^) have higher sensitivity than bismuth-modified paper-based electrodes (0.7 *µ*g ml^−1^).^43^ Yet, all three disposable sensors have not been implemented into real practice.

There has been a focus on sensitivity and selectivity optimization to compare standard methods, such as ICP-MS, and electrochemical sensors. Limit of detection accounts for detection sensitivity and recovery accounts for ensuring a representative measurement of the lead in sample, including the effectiveness of sample preparation steps to account for particulates.^32^ US EPA Method 200.8 indicates a lead detection limit of 0.6 *µ*g l^−1^ for ICP-MS analysis. ICP-MS average lead recovery was determined to be 104 ± 3.42%.^27^ Triantafyllidou et al. (2023) found commercially available electrochemical systems had a limit of detection of 2 *µ*g l^−1^ and lead recovery of 104.9% when comparing to the standard ICP-MS measurement.^44^ Limits of detection have dropped to 0.2 *µ*g l^−1^ for bismuth-coated electrodes.^45^ Other studies have achieved detection limits as low as 0.1 *µ*g l^−1^ on various electrode materials.^34^ Lead in drinking water is often in solution with zinc, copper, cadmium, and calcium. A goal has been to ensure these peaks are distinct, though that has also been achieved.^36^^,^^41^ Sensitivity and selectivity alone do not establish field readiness. Ease of use, negative, and positive response rates are also required. However, as described below, it appears that while there are some technical hurdles, there are further system-level incentives missing that would be required to adopt electrochemical methods as alternatives standards.^27^^,^^46^

Several electrochemical lead sensors have been commercialized as alternatives to standard detection methods and over-the-counter colorimetric test kits. The Palintest Kemio Heavy Metals (Palintest, CO, USA) and Trace2o Leadometer (Trace2o, Berkshire, UK) are examples of portable systems reported to use stripping-voltammetric measurements. The Palintest Kemio Heavy Metals provides real-time, sensitive results, LOD of 1 *µ*g l^−1^ and the Trace2o Leadometer has a LOD of 5 *µ*g l^−1^. Both are below the US EPA action limit. These sensors offer low limits of detection but sample preparation ends with neutralization so often under estimate high particulate lead concentrations or report unacceptable false negatives.^44^ The Palintest Kemio Heavy Metals and Trace2o are not affordable for the average household, but can be affordable for research labs, environmental consulting firms, and the government. These commercial electrochemical sensors are available for real applications but are limited by their inability to account for particulate lead, cost for individual users, and lack of EPA approval.

General concerns and potential unknowns about electrochemical sensors include sample preparation procedures, preservation or digestion where appropriate, reliability of the sensor, sensitivity compared to standard methods, size, weight, portability of supporting equipment, calibration, matrix checks, quality control criteria, operator training, data traceability, and verification. Sample preparation is needed to ensure the total lead, both particulate and dissolved, is being quantified. However, some electrochemical sensors are not compatible with effective lead dissolution solvents. The accuracy of the lead measurement then becomes a concern because only a portion of the total lead concentration is being quantified. Reliability is another concern because in real drinking water samples, electrochemical sensors can be subjected to fouling, matrix effects, stability issues, and calibration complexities.^47^^,^^48^ For example, Jacobia and Jones (2025) showed 87.04 ± 26.52% lead nitrate recovery in test drinking water-based nitric acid using a bismuth-coated glassy carbon electrode and 138.14 ± 13.59% lead nitrate recovery in real tap water samples treated with nitric acid using a bismuth-coated glassy carbon electrode.^49^ Arduini et al. (2010) reviews bismuth-based electrodes that have achieved limits of detection as low as 0.05 *µ*g l^−1^ in lab-based conditions and other studies that have achieved tap water limits of detection of 2.5 ± 0.05 *µ*g l^−1^ with lead recoveries of 118% and 119% in tap water using bismuth-based electrodes.^47^ There is a need for a stable sensor with minimal interfering effects. Supporting equipment for electrochemical sensors includes portable potentiostats, wires, and electrodes. They can be costly, potentially decreasing the economic benefit of shifting away from standard lab-based methods. Therefore, electrochemical sensors have the potential to be complementary to standard methods instead of completely replacing the use of standard methods. However, recent studies have aimed to address particulate lead limitations to improve accuracy electrochemical detection.^49^^,^^50^

Regulation and standardization are equally important challenges to commercialization as the technical challenges.^51^ Adoption of novel technology through government regulation or industry standardization requires meeting the technical challenges, but this adoption also provides a pathway towards profit for manufacturers or environmental testing firms.^52^ Since existing regulations only requires testing the utility side of drinking water, public labs that can afford or apply for funding to afford the existing standard equipment are the only ones setting demand. While there is evidence consumers respond to reports of water quality violations, their potential to respond and induce demand for electrochemical sensors is hindered by many factors.^24^^,^^53^ One pathway forward would be the use of electrochemical methods for high spatial resolution water quality screening and targeted decision making while leveraging existing standard methods for defined high-risk or regulatory decisions making.

## Future Needs and Prospects

Lead paint testing provides a useful model for considering drinking water testing. Federal lead-paint rules established training, certification, and recognition pathways for real estate inspection activities and test methods.^54^^,^^55^ The US EPA enacted the Lead Renovation, Repair and Painting (RPR) Rule which requires sellers or owners to declare lead, and some transactions require testing in places such as homes, daycares, and public buildings.^56^^,^^57^ Results must be reported back to real estate buyers or renters before purchase finalization. Remediation, removal, and cost of cleanup were originally left to the property owner.^58^ In the case of daycares and schools, the government is responsible for initiating cleanup.^58^ Certified assessors and inspectors are hired and use an EPA-recognized lead paint test to determine if home or building conditions comply with EPA lead action limits.

EPA-recognized lead tests are alternative tests to standard methods that have gone through extensive performance analyses to determine detection limits, negative and positive responses, and confidence intervals. Tests must comply with 40 CFR 745.88(c) to become EPA-recognized.^57^ Specifics include negative responses for concentrations greater than or equal to 1.0 mg cm^−2^ or 0.5% by weight with 95% confidence and positive responses for concentrations less than 1.0 mg cm^−2^ or 0.5% by weight with 95% confidence. The EPA’s Environmental Technology Verification Program is responsible for ensuring the test kits comply with the criteria, providing reports of the test kit analyses. Three test kits have been EPA-recognized for lead paint: LeadCheck, D-Lead, and the Commonwealth of Massachusetts kit (Fig. 2). Non-destructive hand-held X-ray fluorescence sensors have been used for lead paint testing and do not require technical interpretation. While the handheld devices are more affordable ($250—$1000), they still require a certified home inspector to satisfy the RPR rule. Other EPA-recognized rapid at-home tests using swabs, strips, wipes, or paint chips as samples require certified assessors to satisfy the RPR and to ensure homeowner trust in the test results. Lead paint testing through the US EPA’s certified laboratory list (40 CFR 142.10(b)(3)(i)) is also an option for homeowners.

Unlike lead paint testing, drinking water testing does not have a regulatory system for private systems. Utilities conduct required monitoring and public reporting using Consumer Confidence Reports (CCRs); however, private line testing remains the responsibility of the property owner and there is no requirement for disclosure at point-of-sale of property.^25^ CCRs assumes utility water quality is the same as the water quality of the home, but that hides the reality.^59^ Private LSL inventories are not extensive enough to support this assumption. Private side drinking water testing is limited to certified drinking water laboratories and alternative test kits that have not been approved by the EPA (Fig. 2). Over-the-counter test kits are available, affordable, and provide rapid results, however, they require user interpretation and are not always accurate.^32^

When lead contamination concerns arise on the private drinking water line, the EPA recommends sending samples to certified laboratories and reaching out to utilities about service line materials. Certified laboratory testing has long processing time, high cost, and requires users to perform sampling and shipping. An EPA website recommends testing with changes to lead contamination risks like observable changes in water quality and changes to the household such as a new baby, pregnancy, or proximity to chemical spills but, this recommendation is not required to be disseminated by public utilities. Dissolved and small particulate lead is colorless, tasteless, and unnoticeable at moderate concentrations in drinking water. Therefore, routine and non-routine water testing should be implemented without consumer recognition.^13^

There are several areas where private-side water quality testing can be improved. The testing water quality testing market has lower profit margins than the rest of the environmental consulting services sector with costs dominated by salary.^60^ A hired certified inspector might still pose challenges like affordability, but, as electrochemical sensors may also be smart enabled devices this may enable broader coverage.^61^^–^^63^ In-home professional testing allows homeowners to have a say in when and how their water is tested without being directly responsible for the physical testing. Certified inspectors also generate results that can contribute to data banks for government and policy use. Increasing available data provides spatial, temporal, and seasonal information on lead contamination and potentially lead service line inventory.

Establishing a role for routine and event-triggered electrochemical testing is not solely a sensor-development challenge. The lead-paint EPA verification model offers useful implementation concepts, but a drinking water pathway must be designed around the distinct exposure pathways, analytical, and sampling challenges of drinking water. For example, a minimum testing frequency is established at the point-of-sale and on a routine basis for schools and daycares for lead paint due to predictable exposure and risk profiles. Establishing testing frequencies for water will have to account for routine chlorination and non-routine events like pregnancies that change both exposure and risk. A potential solution is integrating electrochemical sensors with the water smart grid.^61^^–^^63^

## Conclusions

Lead contamination risk is not distributed equally across locations and communities. Accessible detection methods can help identify risk and efficiently remediate by providing real-time personalized results to consumers and supporting centralized standardized methods such as ICP-MS, ICP-OES, and AAS. While electrochemical lead detection may never replace centralized testing, they could be implemented as an option for routine and non-routine drinking water monitoring. Congress and the EPA have developed a framework that compliments centralized testing and respected property and privacy rights for lead paint testing using portable certified test kits and inspectors for on-site testing. A similar model could be used for lead testing in drinking water. Not only would electrochemical sensors improve accessibility, turn-around time, and trust, but also focus remediation efforts, precautionary measures, and help achieve the federal goal of a comprehensive lead plumbing inventory.
